# Back to the Diving Board: A Rare Cause of Hemoptysis in a Healthy Female Athlete

**DOI:** 10.1155/crpu/5227230

**Published:** 2025-11-28

**Authors:** Mary Blumenfeld, Casey Silvernale, Elena Stekolchik, Seema Rani

**Affiliations:** ^1^Division of Pulmonary and Sleep Medicine, Nemours Children's Health, Wilmington, Delaware, USA; ^2^Sidney Kimmel Medical College at Thomas Jefferson University, Philadelphia, Pennsylvania, USA

**Keywords:** celiac disease, diffuse alveolar hemorrhage, gluten-free diet, idiopathic pulmonary hemosiderosis, Lane–Hamilton syndrome, systemic corticosteroids

## Abstract

Lane–Hamilton syndrome is a rare association between idiopathic pulmonary hemosiderosis (IPH) and celiac disease that can present with isolated hemoptysis. We describe a case of a previously healthy 15-year-old female who presented with 1 month of intermittent hemoptysis, chest pain, and severe iron deficiency anemia. Physical examination was remarkable for mild conjunctival pallor and livedo reticularis. Extensive laboratory studies were obtained to rule out infectious, rheumatological, and oncological explanations of anemia. A chest radiograph showed an increased density in the left mid-lung and bilateral opacities, and computed tomography showed scattered multifocal ground glass opacities. Bronchoalveolar lavage revealed hemosiderin-laden macrophages consistent with the diagnosis of IPH. Additional workup for IPH revealed elevated celiac titers. Upper endoscopy confirmed the diagnosis of celiac disease, which led to a final diagnosis of Lane–Hamilton syndrome, a rare co-occurrence of IPH and celiac disease. The treatment plan included a course of systemic steroids and a gluten-free diet. Given this known association, workup for IPH should include testing for celiac disease since a gluten-free diet may prevent future occurrences of alveolar hemorrhage.

## 1. Introduction

Idiopathic pulmonary hemosiderosis (IPH) is a rare but potentially fatal condition found primarily in children that is characterized by recurrent episodes of diffuse alveolar hemorrhage (DAH) leading to chronic anemia and interstitial fibrosis [1–5]. DAH presents with a syndrome of hemoptysis, anemia, and diffuse radiographic pulmonary infiltrates that can ultimately lead to hypoxemic respiratory failure and permanent lung injury [1–5]. The exact etiology of DAH is unknown; however, it is hypothesized to be an immune-mediated process (e.g., seropositive systemic vasculitides and systemic lupus erythematosus), and there are other nonimmune etiologies of DAH (e.g., coagulopathies, infection, and drug-mediated) [1–5]. IPH as an underlying etiology of DAH is a diagnosis of exclusion with a reported incidence of 0.24–1.23 per 1 million and a 50% mortality rate in the pediatric population [2, 4]. Laboratory and radiographic findings are nonspecific, but the cardinal feature of IPH is the presence of hemosiderin-laden macrophages (HLMs) on bronchoalveolar lavage (BAL) [1–5]. The exact cause of IPH is unknown, but it is hypothesized to be an immune-mediated process, and treatment typically includes systemic corticosteroids [1–5]. Prognosis is variable with a mean survival rate of 2.5–5 years after diagnosis, although there are reports of spontaneous remission [3]. The rarity and nonspecific findings of IPH can lead to a delay in diagnosis and treatment, which can have catastrophic consequences such as death from acute massive hemorrhage or progressive pulmonary insufficiency and right-sided heart failure [3].

## 2. Case Presentation

**Figure 1:**
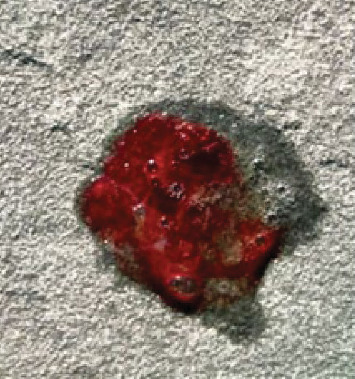
Hemoptysis on the day of presentation.

The patient is a 15-year-old previously healthy female who presented with 1 month of intermittent hemoptysis, chest pain, shortness of breath, and lightheadedness. She initially presented to an outside hospital for evaluation of hemoptysis (Figure 1) and was found to have an oxygen saturation of 90% (on room air) and a critically low hemoglobin of 5.1 g/dL (3.17 mmol/L). Due to the severity of clinical presentation and laboratory findings, she was transferred to a tertiary care children's hospital emergency department for further workup.

**Figure 2:**
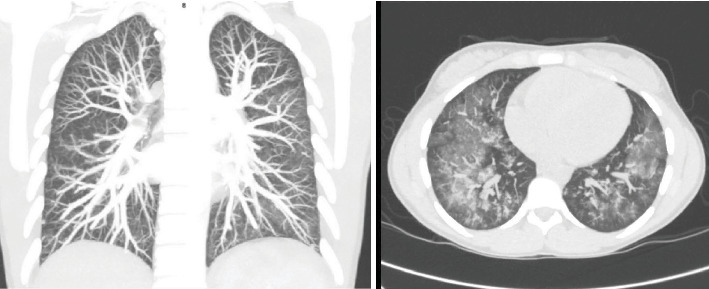
Computed tomography of the chest showing bilateral ground glass opacities.

Additional history revealed that she was diagnosed with multifocal pneumonia based on radiographic findings on chest computed tomography (CT) scan 4 weeks prior to presentation (Figure 2). She was treated with oral antibiotics as an outpatient with no change in clinical symptoms of dyspnea or hemoptysis. She was a competitive springboard diver but denied any chest trauma. She reported tasting and coughing up small specks of blood, in addition to shortness of breath after periods of intense exercising and diving. She had no history of risk factors for tuberculosis or recent travel. There was no history of vaping, cigarette smoking, or other drug use. There was no prior history of asthma, bronchitis, pneumonia, or other chronic respiratory or gastrointestinal symptoms. She had never been on inhaled medications.

On presentation, the patient was well-appearing and in no acute distress. She had mild conjunctival pallor but no facial pallor, with normal respiratory effort and clear lung sounds bilaterally. Faint livedo reticularis was visible on bilateral thighs, but there were no petechiae, purpura, or other rashes, and no edema was appreciated. Laboratory studies showed microcytic anemia and low iron (Table 1), indicating severe iron deficiency anemia likely secondary to chronic blood loss. Blood transfusion was completed with the guidance of hematology clinicians, given the severity of anemia. Multiple subspecialties, such as pulmonology, hematology/oncology, rheumatology, and infectious diseases, were involved. There was no family history of autoimmune diseases such as juvenile idiopathic arthritis, systemic lupus erythematosus, vasculitis, inflammatory bowel disease, celiac disease, thyroid disease, or psoriasis.

**Figure 3:**
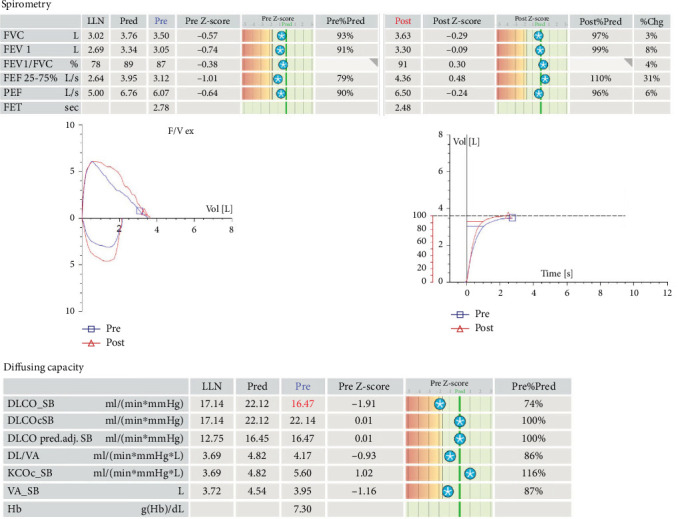
Spirometry testing and diffusion capacity testing.

Additional infectious and inflammatory workup (Table 2) did not reveal an underlying cause of her symptoms. Further testing of pulmonary function including 6-min walk test, diffusion capacity of the lungs for carbon monoxide corrected for alveolar volume, and spirometry was normal (as shown in Figure 3). The airflows were normal without evidence of airway obstruction or bronchodilator reversibility. A CT scan of the chest demonstrated patchy multifocal ground glass opacities (Figure 2). Due to the clinical history and abnormal radiographic imaging, flexible bronchoscopy was pursued.

**Figure 4:**
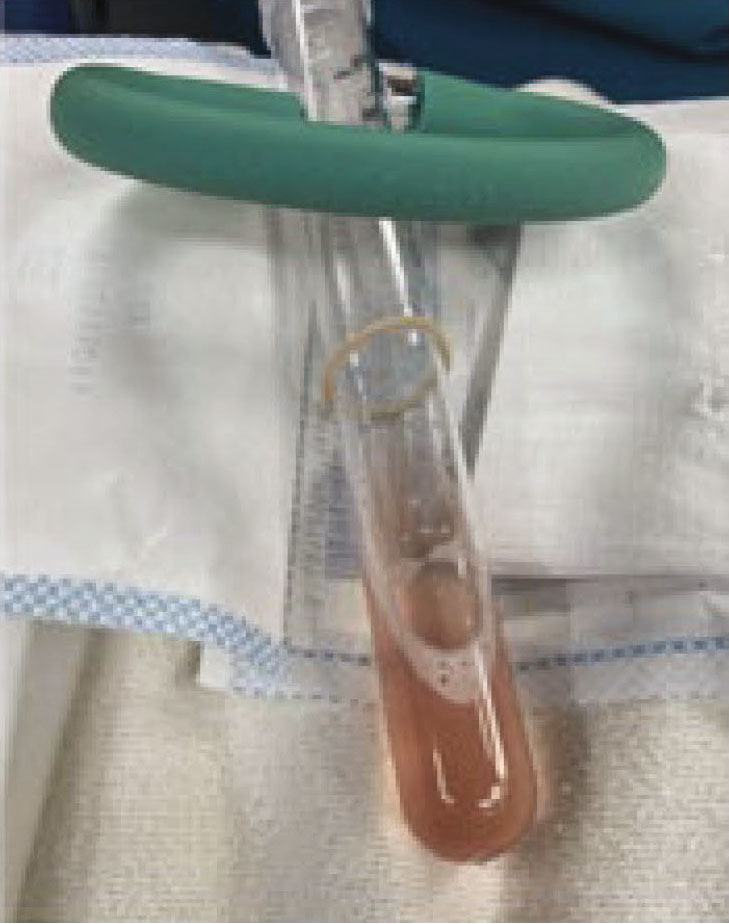
Aspirate from the patient's initial bronchoalveolar lavage showing pink-tinged return indicating diffuse alveolar hemorrhage.

**Figure 5:**
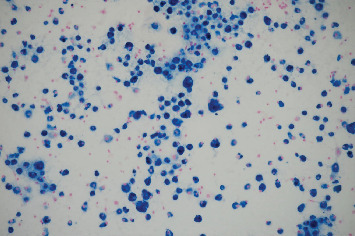
Prussian blue stain positive for iron in macrophages (January 2023).

**Figure 6:**
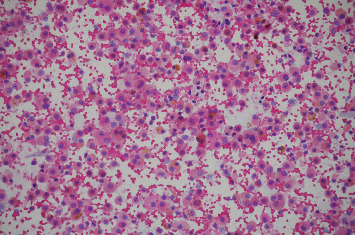
Pathology slide: hematoxylin and eosin stain from the patient's bronchoalveolar lavage showing 50 per 40x power field of hemosiderin-laden macrophages (January 2023).

Bronchoscopy revealed normal anatomy, normal mucosal lining of the tracheobronchial tree, and normal secretions with no evidence of frank blood in the airways. BAL fluid was pink-tinged, which was suggestive of DAH (Figure 4). The pathology report revealed numerous red blood cells, pigment-laden macrophages, and scattered ciliated bronchial epithelial cells. Iron stain showed numerous (50 per 40x power field) HLMs (Figures 5 and 6) consistent with pulmonary hemosiderosis. Bacterial, fungal, and mycobacterial cultures were negative.

Based on bronchoscopy findings and multidisciplinary investigation for causes of pulmonary hemorrhage, IPH was suspected. Celiac testing was completed as part of the workup for IPH, which showed elevated tissue transglutaminase and endomysial antibody raising suspicion for celiac disease (Table 3). There were no reported gastrointestinal symptoms such as weight loss, abdominal pain, diarrhea, or specific food intolerance. Lane–Hamilton syndrome (LHS) was suspected based on radiographic findings, pathology studies of BAL, and abnormal celiac testing.

**Figure 7:**
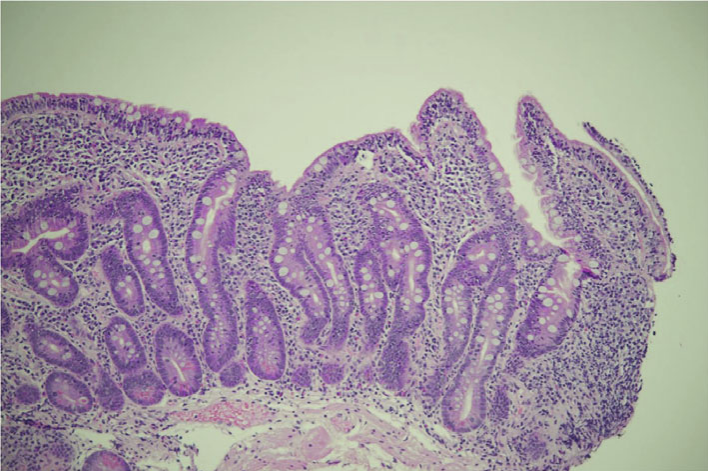
Duodenal biopsy with villous blunting and increased intraepithelial lymphocytes.

LHS is a rare co-occurrence of IPH and celiac disease. Gastroenterology was consulted in the outpatient setting due to the abnormal celiac panel (elevated tissue transglutaminase IgA and positive endomysial antibody) and recommended endoscopy, which showed a grossly normal stomach with biopsies revealing mild, nonspecific chronic gastritis. The duodenum had gross changes of scalloping and flattening of folds, and biopsies revealed villous blunting with foci of intraepithelial lymphocytes and intraepithelial neutrophils, which confirmed the diagnosis of celiac disease (as shown in Figure 7).

**Figure 8:**
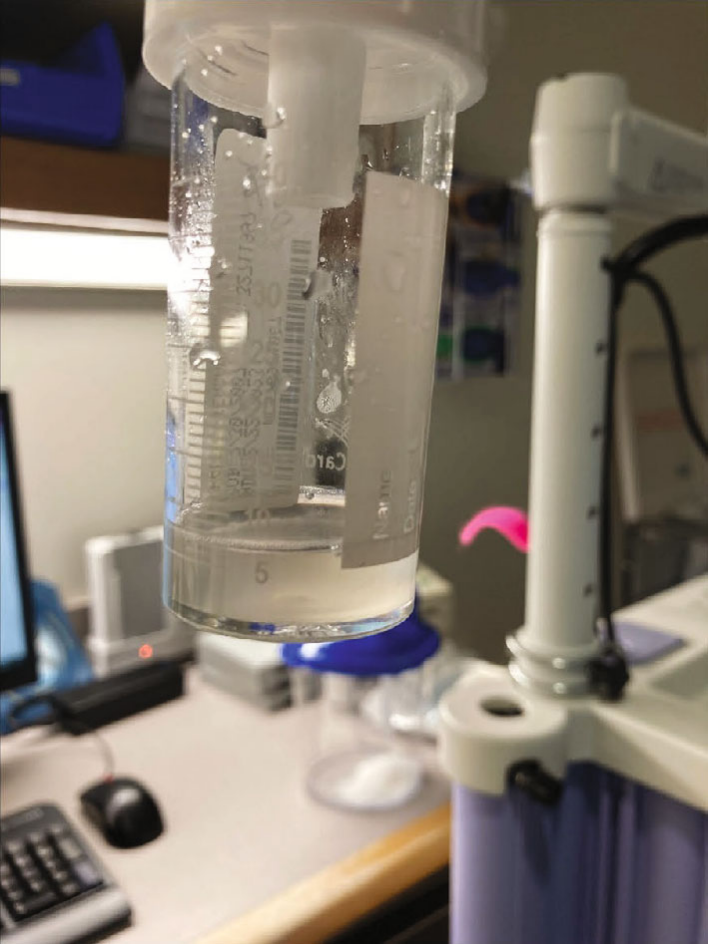
Bronchoalveolar lavage fluid aspirate from the second bronchoscopy (following a 21-day steroid course in February 2023).

**Figure 9:**
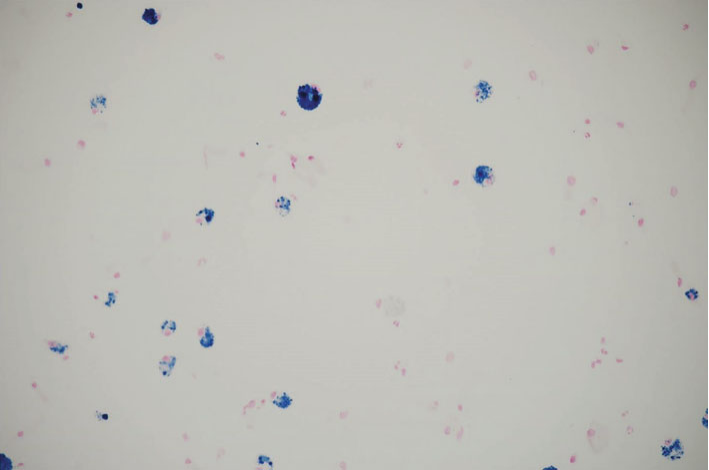
Prussian blue stain positive for iron in macrophages but demonstrating significant reduction (17 per 40x power field) in iron-stained macrophages (poststeroid course done on bronchoalveolar lavage in February 2023).

**Figure 10:**
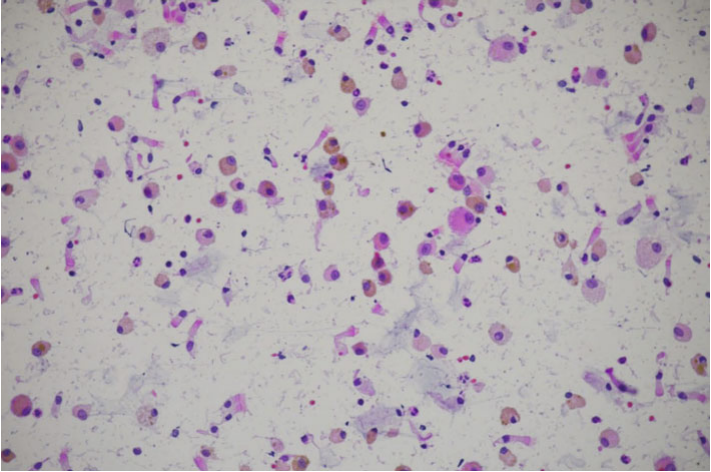
Pathology slide: hematoxylin and eosin stain from the patient's bronchoalveolar lavage poststeroid course showing significant reduction in hemosiderin-laden macrophages (poststeroid course bronchoalveolar lavage done in February 2023).

Repeat bronchoscopy was performed at the time of endoscopy following a 21-day course of systemic steroids. Bronchoscopy revealed normal anatomy, mucosa, and secretions, but BAL return was clear in contrast to her previous BAL (Figure 8). Pathology further revealed a significant reduction in HLMs (Figures 9 and 10).

**Figure 11:**
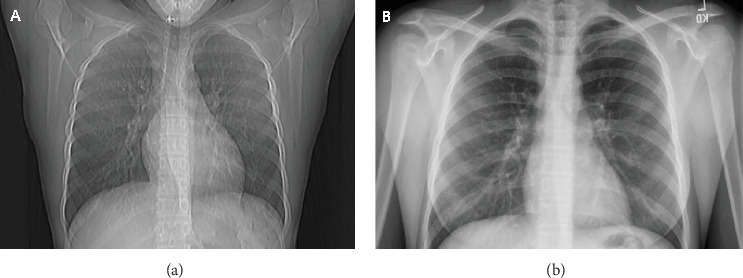
(a) Initial chest radiograph showing bilateral hazy airspace opacities. (b) Repeat chest radiograph following a 21-day steroid taper showing resolution of bilateral hazy airspace opacities.

A gluten-free diet was initiated following endoscopy. Repeat chest radiograph demonstrated significant improvement of bilateral opacities (Figure 11). There was resolution of respiratory symptoms including hemoptysis, and she resumed competitive diving. Additionally, anemia resolved with repeat hemoglobin of 13.1 g/dL (8.13 mmol/L) 7 weeks following initial hemoglobin of 5.1 g/dL (3.17 mmol/L). The family was counseled on the importance of strict adherence to the gluten-free diet in an effort to prevent exacerbation of this condition.

## 3. Discussion

Multiple other differential diagnoses were considered including autoimmune disorders like systemic lupus erythematosus (which can be associated with pulmonary capillaritis as well as hemolytic anemia), antineutrophilic cytoplasmic autoantibody vasculitis, and granulomatosis with polyangiitis (formerly called Wegener's granulomatosis), but workup was negative. Antinuclear antibodies and antineutrophilic cytoplasmic autoantibodies were elevated but were determined to be cross-reactive as the reflex specific titers were negative. Elevated celiac titers led to a final diagnosis of LHS, a rare co-occurrence of IPH and celiac disease. There have only been 80 reported cases of LHS worldwide since 1971, more than half of which were in children [6]. The most common presentations include hemoptysis, cough, dyspnea, and iron deficiency anemia [2]. Isolated hemoptysis and cough can be mistaken for a respiratory tract infection, leading to an inaccurate or delayed diagnosis. It is important to have a thorough differential diagnosis for hemoptysis, including workup for celiac disease, if an underlying etiology is not identified. In the majority of cases, IPH and celiac disease present concurrently; however, an isolated presentation of IPH followed by celiac disease has been reported. IPH can also occur as episodic flare-ups in cases with existing celiac disease. According to some studies, when not diagnosed concurrently, the lag time of other pathology either celiac disease or IPH was about 2.5 years (min/max: 3 months/11 years) leading to a delayed diagnosis [2]. LHS is a medical emergency with a 14% mortality rate due to hemorrhage in acute IPH [7]. Patients can present in respiratory failure and hypoxemia requiring intensive management, and in some cases, LHS has been associated with death [3–5]. Thus, patients with a clinical suspicion for IPH should be screened promptly for celiac disease with serologic testing, such as tissue transglutaminase IgA and endomysial antibodies, even in the absence of gastrointestinal symptoms [1, 2, 4–8]. Some correlation studies have demonstrated that recurrent episodes of hemoptysis were associated with diarrhea in those with LHS. Definitive diagnosis of celiac disease requires duodenal biopsy. It is not understood why children with LHS do not typically present with gastrointestinal symptoms, and the cause of pulmonary hemorrhage is thought to be immune-mediated. Some pathophysiological mechanisms have been postulated in LHS including T-cell–mediated immune response stimulated by gluten and circulating immune complex stimulated by food allergens in the alveolar basal membrane or inhalation of gluten, which can trigger an immune complex formation [6, 7]. Chest radiographs can be normal, so CT should be done in suspected cases. CT typically demonstrates bilateral patchy infiltrates and ground glass opacities.

Although lung biopsy is the gold standard for diagnosing IPH, this is very invasive. Flexible bronchoscopy and BAL, which demonstrate HLMs, are pathognomonic for alveolar hemorrhage and aid in diagnosing IPH in the majority of cases. BAL cytology, in some cases, demonstrates a decreased CD4/CD8 ratio, which has been shown to be normalized after 1 month of a gluten-free diet. Pulmonary function testing when performed is often normal in most cases. Active hemorrhage in IPH can result in an increase in lung diffusion capacity for carbon monoxide; however, in chronic cases where there is thickening of alveolar septa, there could be a reduction in lung diffusion capacity for carbon monoxide along with a restrictive pattern over time. Systemic corticosteroids and pulse steroids are administered in the majority of cases during acute presentations, in addition to other acute management like blood transfusion and iron supplementation. A gluten-free diet is recommended if the diagnosis of celiac disease is suspected. Frequent exacerbations are treated with pulse corticosteroid therapy, in addition to other steroid-sparing agents such as azathioprine and hydroxychloroquine. Systemic steroids and agents such as azathioprine, hydroxychloroquine, and mycophenolate mofetil remain the gold standard for treating DAH. However, a gluten-free diet may prevent recurrence of DAH and reduce the need for long-term corticosteroids or other immunosuppressants in patients with LHS and is the only effective long-term therapy for all ages [8].

## Figures and Tables

**Table 1:** Initial complete blood count and iron panel status post red blood cell transfusion.

**Measure (reference range and units)**	**Value**
White blood cell count (4.5–11.5 K/*μ*L [4.5–11.5·10^9^/L])	5.5
Hematocrit (36%–46%)	22 (L)
Hemoglobin (12.0–16.0 g/dL [7.45–9.93 mmol/L])	6.0 (L)
Red blood cell count (4.1–5.1 M/*μ*L [4.1–5.1·10^12^/L])	3.51 (L)
Mean corpuscular volume (78–102 fL)	63 (L)
Mean corpuscular hemoglobin (25–35 pg [1.5514–2.1720 fmol])	17 (L)
Mean corpuscular hemoglobin concentration (31–37 g/dL [19.2379–22.9614 mmol/L])	27 (L)
Red cell distribution width (11.5%–15.0%)	22.8 (H)
Reticulocyte count	0.7
Iron (28–184 *μ*g/dL [5.01–32.94 *μ*mol/L])	27 (L)
Total iron binding capacity (260–445 *μ*g/dL [46.54–79.66 *μ*mol/L])	444
Iron saturation percentage	6
Ferritin (10.00–70.00 ng/mL [10–70 *μ*g/L])	7.77 (L)

*Note:* H, high; L, low.

**Table 2:** Inflammatory workup.

**Measure (reference range and units)**	**Value**
Erythrocyte sedimentation rate (0–20)	14
C-reactive protein (0.0–0.9)	< 0.5
Rheumatoid factor (< 14)	< 14
Antinuclear antibody (< 40)	80 (H)
Antineutrophil cytoplasmic antibodies (< 20)	40 (H)
Double-stranded DNA (0–200)	105
Beta-2 glycoprotein immunoglobulin G (< 20.0)	< 2.0
Beta-2 glycoprotein immunoglobulin M (< 20.0)	3.7
Complement component 3 (111–177)	99 (L)
Complement component 4 (9–36)	22
Dilute Russell's viper venom time screen ratio (< 1.21)	0.99
Anti-myeloperoxidase	Negative
Proteinase 3 antibody	Negative

*Note:* H, high; L, low.

**Table 3:** Celiac workup.

**Measure (reference range)**	**Value**
Endomysial antibody titer (< 1:5)	1:320
Endomysial immunoglobulin A antibody (negative)	Positive
Tissue transglutaminase immunoglobulin A (0–3 U/mL)	> 100 (H)
Tissue transglutaminase immunoglobulin G (0–5 U/mL)	21 (H)

*Note:* H, high.

## Data Availability

Data are contained within the manuscript.

## Nomenclature

BAL: bronchoalveolar lavage
CT: computed tomography
DAH: diffuse alveolar hemorrhage
HLMs: hemosiderin-laden macrophages
IPH: idiopathic pulmonary hemosiderosis
LHS: Lane–Hamilton syndrome
